# Should providers encourage realistic weight expectations and satisfaction with lost weight in commercial weight loss programs? a preliminary study

**DOI:** 10.1186/2193-1801-3-477

**Published:** 2014-08-28

**Authors:** Gretchen E Ames, Colleen S Thomas, Roshni H Patel, Jillian S McMullen, Lesley D Lutes

**Affiliations:** Obesity Medicine, Division of General Surgery, Mayo Clinic, 4500 San Pablo Road, Jacksonville, FL 32224 USA; Department of Health Sciences Research, Mayo Clinic, 4500 San Pablo Road, Jacksonville, FL 32224 USA; Department of Psychology, East Carolina University, 1001 E 5th St., Greenville, NC 27858 USA

**Keywords:** Weight loss expectations, Attrition, Obesity treatment

## Abstract

**Background:**

Attrition is a problem among patients who participate in commercial weight loss programs. One possible explanation is that if patients are unable to reach a weight that they expect to achieve, they may be more likely to drop out of treatment. This study investigated variables associated with attrition among 30 obese patients who completed a liquid meal replacement program (LMR) and enrolled in a 52-week Small Changes Maintenance intervention (SCM). Patients lost a median 18% of body weight during LMR and completed assessments about weight expectations and weight satisfaction pre- and post-SCM.

**Findings:**

Of the 30 patients who started SCM, 8 (27%) were lost to attrition. Odds of SCM attrition were higher in patients who lost ≤ 18.2% of pre-LMR weight (OR: 12.25, P = 0.035), had lower satisfaction (≤7) pre-SCM (OR: 10.11, P = 0.040), and who expected further weight loss of 9.1 kg or more pre-SCM (OR: 10.11, P = 0.040). SCM completers significantly increased weight loss expectations by a median of 2.3 kg from pre-SCM to post-SCM (WSR P = 0.049) that paralleled weight regained post-SCM (2.7 kg).

**Conclusions:**

After completion of a medically-supervised commercial weight loss program, patients with the greatest expectations for further weight loss and the lowest weight satisfaction were more likely to drop out of SCM. Failure to participate in maintenance treatment may lead to regain of greater than half of lost weight over the next year. Among SCM completers, lower expectations for further weight loss and greater weight satisfaction appeared to be associated with continued engagement in maintenance treatment.

## 1. Introduction

Patients seeking weight loss treatment frequently set goals and expectations of losing ≥25% of their initial body weight (Foster et al. 1997; Wadden et al. 2003). Weight loss of this magnitude greatly exceeds the typical weight loss achieved during behavioral treatment. A weight goal is defined as the amount of weight patients would ideally like to lose during treatment (Fabricatore et al. 2008; Fabricatore et al. 2007) whereas a weight loss expectation is defined as how much weight patients think they can lose during treatment. Weight loss expectations tend to be more moderate than weight goals (e.g., ultimate or dream weight) although they are still significantly greater than the 5-10% reduction in initial body weight that is typically achieved with behavioral treatment (Fabricatore et al. 2007). To date, studies examining the influence of weight expectations have yielded mixed results. In the short-term, unrealistic weight expectations do not appear to have deleterious effects for patients participating in controlled clinical trials (Ames et al. 2005; Fabricatore et al. 2007; Moore et al. 2011; Wadden et al. 2003). However, much less is known about the long-term effects when weight loss falls short of initial expectations for patients who are paying for treatment in “real world” settings (Dutton et al. 2010).

One potential problem is that if patients are unable to reach a weight that they expect to achieve, they may be more likely to drop out of treatment. Previous research has shown that higher expectations for weight loss at baseline among patients seeking a variety of obesity treatments in a medical setting (e.g., nutrition education, cognitive behavioral therapy, medication) were associated with higher rates of attrition from treatment at 12-months. (Dalle Grave et al. 2005). A significant problem related to attrition is treatment failure. In other words, when patients discontinue participation in treatment, they are significantly more likely to regain lost weight (Ames et al. 2014). Further research is needed to determine what variables are associated with attrition such as weight loss expectations and satisfaction with weight in medical settings where patients are paying for treatment (Dalle Grave et al. 2005). Weight loss expectations, among other variables, could potentially be targeted for intervention in an effort to reduce rates of attrition.

This study was conducted during an investigation of a 52-week Small Changes Maintenance intervention (SCM) for patients who had completed a medically-supervised commercial liquid meal replacement program (LMR). Details about SCM have been described previously (Ames et al. 2014). The present study had two primary goals. First, based on findings from a previous research (Dalle Grave et al. 2005), we hypothesized that specific patient variables would be associated with attrition from SCM. Those variables included percent of initial weight lost during LMR, satisfaction with weight pre-SCM, expectation for further weight loss pre-SCM, and duration of time expected to achieve desired weight loss. Second, we were interested in investigating if patients’ expectations for further weight loss would decrease after 52-weeks of maintenance treatment. We hypothesized that expectations for further weight loss would decrease to reflect actual body weight after patients attempted to sustain weight lost during LMR for 52 weeks.

## 2. Methods

### 2.1 Study patients

The study included 30 consecutive patients who completed a 21-week medically- supervised 800 calorie (OPTIFAST 800®) LMR and enrolled in the 52-week SCM. Inclusion and exclusion criteria for enrollment in SCM have been described previously (Ames et al. 2014). The median age of the 30 patients who completed LMR and enrolled in SCM was 60 years (range, 22 to 77 years). Patients were predominately female (67%) and white (83%). Pre-LMR, the median BMI was 40.9 kg/m^2^ (range, 25.4 to 63.9 kg/m^2^) and median body weight was 111 kg (range, 72 to 178 kg). Patients lost a median of 18% (range, 10 to 32%) of pre-LMR weight. The study was approved by the medical center institutional review board.

### 2.2 Procedures

Data collection for this study occurred during SCM that employed a one-group treatment design and a historical comparison group (Ames et al. 2014). Patients received 20 treatment sessions over a period of 52 weeks. Briefly, SCM is theoretically- based program that promotes self-selected changes in behavior (Lutes et al. 2013; Lutes et al. 2008). SCM offered no prescribed changes or preset goals for weight maintenance behaviors and all changes in caloric intake and physical activity were self-selected by the patients. At the time of enrollment in SCM, each patient attended a 45-minute individual counseling session with a registered dietitian as part of routine clinical care. During this visit, all patients were encouraged to focus on weight maintenance and were informed that they should not expect to achieve further weight loss. Survey questions were completed at the conclusion of the visit with the registered dietitian (pre-SCM) and again at week 52 (post-SCM).

### 2.3 Measures

The following questions, derived from previous studies (Fabricatore et al. 2007; Foster et al. 2004), were used to assess weight expectations and satisfaction with weight.

#### 2.3.1 Survey questions

1. Please rate your overall satisfaction with your current weight (1 = “not at all satisfied”, “9 = extremely satisfied”). 2. If you are trying to lose more weight, how many more pounds do you think you can lose (0, 5, 10, 15, 20, 25, 30, 40, or 50 lbs.)? 3. How many months do you think it will take to reach this weight (1 to 12 months)?

#### 2.3.2. Body weight

Patients were weighed in kg in light clothing with shoes off using a Scale-Tronix 6002 wheelchair auto zero scale pre- and post-SCM.

### 2.4. Statistical analysis

The proportion of patients lost to attrition (dropped out of SCM) was estimated along with an exact binomial 95% confidence interval (CI). Associations with SCM attrition were explored using Fisher’s exact tests where the variables of interest (percent of initial weight lost during LMR, satisfaction with weight pre-SCM, expectation for further weight loss pre-SCM, and length of time expected to achieve desired weight loss) were categorized based on the sample median. Odds ratios (OR) for SCM attrition and corresponding 95% CI were estimated. Multivariable logistic regression was not performed owing to the limited number of events observed in this study (only 8 patients were lost to attrition); therefore, results from this study should be considered preliminary. Changes in weight loss expectations pre- to post-SCM were evaluated using a nonparametric Wilcoxon signed-rank (WSR) test among those participants who completed SCM. Two-sided p-values < 0.05 were considered statistically significant. All analyses were performed using SAS statistical software (version 9.3; SAS Institute Inc.; Cary, NC).

## 3. Results

### 3.1. SCM attrition

Of the 30 patients who started SCM, 8 (27%) were lost to attrition prior to the week 52 assessment (95% CI: 12 to 46%). Comparisons in SCM attrition rates between patient groups are summarized in Table 1**.** Odds of SCM attrition were higher in patients who lost ≤ 18.2% of pre-LMR weight (OR: 12.25, P = 0.035), had lower satisfaction (≤7) pre-SCM (OR: 10.11, P = 0.040), and who expected further weight loss of 9.1 kg or more pre-SCM (OR: 10.11, P = 0.040). There was no evidence of an association of the number of months expected to reach their weight loss goal with SCM attrition (P = 1.00).

**Table 1:** **Comparison of attrition rates during the Small Changes Maintenance intervention (SCM) according to pre-SCM variables**

Pre-SCM Variables ^a^	Fraction (%) of patients lost to attrition	OR (95% CI)	P-value ^b^
Percent of weight lost during LMR			0.035
≤18.2%	7/15 (47%)	12.25 (1.27-118.36)	
>18.2%	1/15 (7%)	1.00 (reference)	
Expected weight loss			0.040
0-6.8 kg	1/14 (7%)	1.00 (reference)	
9.1-22.7 kg	7/16 (44%)	10.11 (1.05-97.00)	
Number of months expected to achieve weight loss goal^c^			1.00
1-9	4/14 (29%)	1.47 (0.26-8.23)	
10-12	3/14 (21%)	1.00 (reference)	
Weight satisfaction			0.040
1-7	7/16 (44%)	10.11 (1.05-97.00)	
8-9	1/14 (7%)	1.00 (reference)	

*Abbreviations:* *SCM* Small Changes Maintenance, *OR* odds ratio, *CI* confidence interval, *LMR* liquid meal replacement.

^a^Grouping of pre-SCM characteristics was based on the sample median.

^b^P-values result from Fisher’s exact test owing to the small number of events.

^c^Two patients did not expect to lose more weight.

### 3.2. Weight expectations pre- and post-SCM

**Figure 1:**
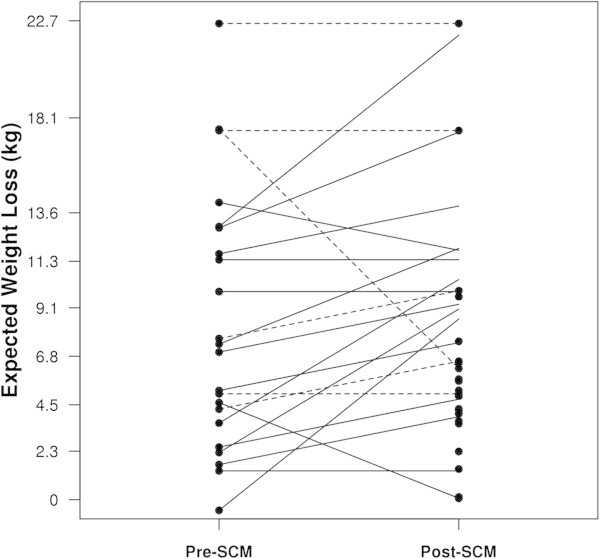
**Changes in expected weight loss before and after the Small Changes Maintenance intervention (SCM) among 22 participants who completed SCM.** *Solid lines represent the 16 participants who gained weight and dashed lines represent the 6 patients who either maintained their weight or lost weight during the 52-week intervention. To minimize overlap of data points, expected weight loss was jittered vertically (within .9 kg) by the same amount for pre- and post SCM.*

Overall, patients (N = 30) expected to lose an additional 10% of body weight (median 9.1 kg.) pre-SCM. Greater than half of SCM completers (N = 22) reported an increase in their expectations for further weight loss (59%), while 27% reported no change in expectation, and only 14% decreased their expectations for further weight loss at week 52 of SCM. SCM completers had significantly increased weight loss expectations by a median of 2.2 kg from pre-SCM to post-SCM (WSR P = 0.049), yet had regained a median of 2.3 kg (14%) of lost weight. Among the 16 patients who gained weight during the intervention, weight loss expectations increased by a median of 2.2 kg (WSR P = 0.016) while they regained a median 5 kg. However, the median change in expected weight loss for the 6 patients who maintained or lost weight during the intervention was 0 kg (WSR P = 1.00) while they lost a median of 1.4 kg. Figure 1 shows weight loss expectations pre- and post-SCM for treatment completers.

## 4. Discussion

Results from this study yielded some important findings about attrition for patients who completed medically-supervised commercial LMR and enrolled in a 52-week SCM. First, patients who lost the most weight during LMR and reported higher ratings of satisfaction with weight pre-SCM were significantly more likely to complete SCM. Conversely, patients who had greater expectations for further weight loss were significantly less likely to complete SCM. This finding is similar to what was found in a previous investigation where greater weight loss expectations before starting treatment were associated with attrition for patients seeking treatment in a medical setting (Dalle Grave et al. 2005). Attrition is a significant problem for LMR patients in particular as failure to participate in follow-up care may lead to greater than 50% regain of lost weight over the next year (Ames et al. 2014). Thus, improving satisfaction with weight during LMR and encouraging moderation of expectations for further weight loss may help patients remain engaged in maintenance treatment thereby attenuating weight regain.

Second, given the large amount of weight they had already lost (18% of initial weight), patients were informed that they should not expect to lose more weight and were encouraged to focus on maintenance of weight lost during LMR. In spite of this recommendation, at the start of SCM patients’ expected to lose an additional 10% of their weight over the next 9.5 months. However, an encouraging finding from this study was that SCM completers, even though they expected some further weight loss, experienced minimal weight regain during the intervention and remained engaged in treatment. Moreover, SCM completers had expectations for further weight loss that did not reflect actual outcome after 52-weeks of treatment. The majority of SCM completers (73%) regained weight yet significantly increased expectations for future weight loss that paralleled weight regained at week 52 of SCM (2.3 kg. vs. 2.7 kg. respectively). Although no definitive conclusions can be derived from this preliminary study, findings suggest that if patients have some expectation for further weight loss, this may minimally influence engagement in maintenance treatment.

This study had several limitations primarily a small sample size resulting in limited statistical power and preliminary findings. Additionally, the sample was comprised of older white patients who had financial resources to participate in the program which may limit the generalizability of the results. Nevertheless, results from this study revealed that greater weight loss expectations and lower satisfaction with weight were associated with attrition from maintenance treatment in a medically-supervised commercial weight loss program. Therefore, patients with the greatest weight loss expectations may benefit from moderation of what weight they expect to achieve and maintain in an attempt to reduce the risk of attrition. Strategies for improving weight satisfaction such as focusing on improvements in health status and mobility may also help reduce the risk of attrition. Future research is needed to replicate these findings in a larger more diverse sample and should compare rates of attrition among patients with high and low expectations for future weight loss. Moreover, rates of attrition should be compared among patients who receive intervention to moderate weight expectations and to improve weight satisfaction with patients who receive usual care.
